# Improvement to the Carrier Transport Properties of CdZnTe Detector Using Sub-Band-Gap Light Radiation

**DOI:** 10.3390/s19030600

**Published:** 2019-01-31

**Authors:** Xiangxiang Luo, Gangqiang Zha, Lingyan Xu, Wanqi Jie

**Affiliations:** State Key Laboratory of Solidification Processing, and MIIT Key Laboratory of Radiation Detection Materials and Devices, Northwestern Polytechnical University, Xi’an 710072, China; fuxu_mail@yeah.net (X.L.); zha_gq@nwpu.edu.cn (G.Z.); xulingyan@nwpu.edu.cn (L.X.)

**Keywords:** semiconducting II–VI materials, photon counting, count rate, point defects

## Abstract

The effects of sub-band-gap light radiation on the performance of CdZnTe photon-counting X-ray detectors were studied using infrared light with different wavelengths in the region of 980–1550 nm. The performance of the detectors for X-ray detection was improved by the radiation of infrared light with the wavelengths of 1200 nm and 1300 nm. This was because the increase of the electron indirect transition, and the weakening of the built-in electric field induced by the trapped holes, reduced the drift time of the carrier, and increased the charge collection efficiency. To further analyze the intrinsic behavior of the trapped charge, the deep-level defects of CdZnTe crystal were measured by thermally stimulated current spectroscopy (TSC). The deep-level defect indicated by the trap named T4 in TSC spectra with the ionization energy of 0.43 eV should be responsible for the performance deterioration of CdZnTe detectors.

## 1. Introduction

Photon-counting energy-dispersive detectors have become popular in the field of X-ray imaging [1,2,3]. By using these types of detectors, the X-ray spectrum can be separated into different energy regions, and the photon numbers in each region can be counted independently [4,5]. Therefore, the attenuation coefficients for different photon energies can be obtained [6,7]. The single crystalline CdZnTe (CZT) semiconductor has created important applications in the field for hard X-ray and γ-ray imaging devices and spectrometers, which have excellent energy and spatial resolutions at room temperature. However, commercial CdZnTe crystals contain high densities of carrier captures, recaptures, and composite defects. External light radiation has been found to be a useful way to stimulate the captured carriers to improve the detector performance [8,9,10,11]. Scientists have investigated the physical mechanisms in relation to the bias induced polarization and the radiation induced polarization [12,13,14,15,16]. Several works used optical perturbations with both sub-band-gap and above-band-gap light sources to enhance the performance of CdTe/CZT detectors. Generally, the above-band-gap light sources reduce the effects of bias induced polarization, while the sub-band-gap light sources reduce the effects of radiation induced polarization [17]. Several studies utilized visible light, laser, and other sub-band-gap light of different wavelengths as the radiation sources.

In this study, sub-band-gap light radiation was adopted to stimulate defect-trapped charges to test the possibility of improving the device properties and to understand the underlying principles. The distribution of crystalline defects in photon-counting CZT detectors with different properties is characterized by thermally stimulated current spectroscopy (TSC) combined with gamma-ray energy analysis. The spectral response corresponds to the carrier mobility–lifetime product. It reveals the effects of different defect energy levels on the detector count, energy spectrum, and overall performance of the CZT photon-counting X-ray detectors.

## 2. Experimental Methods and Results

The effects of sub-band-gap light radiation on the counting properties of the detector were studied by testing the change in the count rate of the detector under the infrared light of different wavelengths. The photocurrent and opt mechanical spectroscopy were measured under different wavelengths of sub-band-gap light in a self-designed setup. The crystal defects were characterized with thermally stimulated current (TSC) spectroscopy.

### 2.1. Studies on the Deep-Level Defects Using TSC

TSC testing was applied to high-impedance indium doped CdZnTe crystals, as a useful method for characterizing deep-level defects. In the experiment, two CdZnTe crystals, one with good and one with poor counting properties were selected. Both CdZnTe crystals were polished, washed, and etched. Au electrode was then prepared by heating AuCl_3_ solution painted on both the sides of CdZnTe crystals. The gold electrode was protected by a photoresist. After the passivation, the so-called CdZnTe detectors for the test were obtained.

The CdZnTe detector was fixed in the chamber of a self-designed TSC system. After electrical contact was connected, the chamber was vacuumed. A liquid helium refrigerator was used to reduce the temperature of CdZnTe detector to 15 K. The detector was irradiated with monochromatic light of wavelength 650 nm, and a bias voltage of 1 V was applied to the detector. Non-equilibrium carriers generated by the external light were trapped by the defect to fill the levels. The light and the dark photocurrent curves were observed for a period of time. The light source was turned off when the trap level filled sufficiently to reach the equilibrium state, i.e., the current became stable. Then, a voltage of 10 V was applied to the detector, and the detector was heated at a constant heating rate to 315 K to obtain a current profile of CdZnTe crystal under light irradiation. Afterwards, the crystal temperature was reduced to 15 K and then heated to 315 K at the same heating rate, so that the dark current spectrum of CdZnTe crystal was obtained for comparison. The current spectrum under light irradiation was subtracted from the dark current spectrum to obtain the defect carrier.

**Figure 1:**
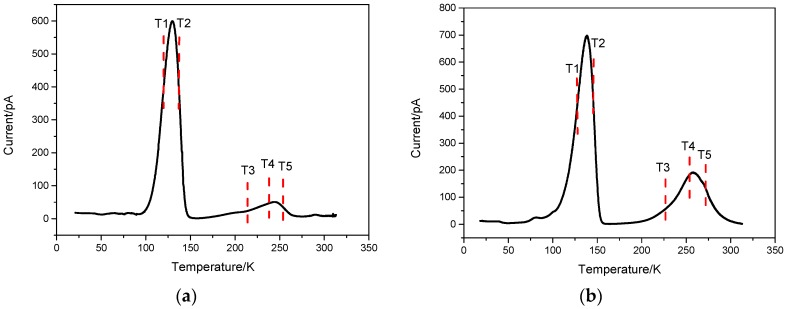
Results of TSC measurement (**a**) detector with better counting performance; (**b**) detector with poor counting performance.

Figure 1 and Table 1 show TSC results obtained at the heating rate of 0.2 K/s. It can be seen that there are five distinct current peaks (or traps) in each chromatogram of CdZnTe detectors, namely T1, T2, T3, T4 and T5. The current peaks correspond to defect states in CdZnTe bandgap. The heat shock current spectrum was decomposed by simple simultaneous multiple peak analysis (SIMPA) and the defect energy was obtained. The levels T1 to T5 are explained as follows:

T1 with the activation energy of 0.05 ± 0.01 eV is a shallow donor defect level, which is introduced by Indium doping, where the interstitial atom of In replaces the Cd vacancies. This energy level was also predicted by the first-principles calculation to be 0.04 eV, below the conduction band [18,19].

T2 located above the valence band is a shallower acceptor defect level with the energy of 0.11 ± 0.02 eV. This defect level is related to Cd vacancy or composite defect caused by Cd vacancy [20,21]. According to the first-principles calculations, the activation energy of the primary ionization Cd vacancy is 0.13 eV.

T3 with the energy level depth of 0.18 ± 0.03 eV is from electron traps and is considered to come from dislocations and their extended defects. When the sample is irradiated, the atom is easily dislocated from this kind of defect, and the defect energy level will increase to some extent [16,22,23].

T4 located at 0.42 ± 0.08 eV above the valence band is from deep hole traps. According to the first-principles calculation, T4 defect level is introduced by the secondary ionized Cd vacancy [24,25].

T5 located at 0.58 ± 0.1 eV above the valence band is a deep electronic defect. The first-principles calculation shows that the activation energy of the counter ion of the secondary ionization Te is 0.59 eV, which is very close to the energy corresponding to T5. Therefore, it is considered that the level is related to the anti-sites of the secondary ionization Te [26,27].

It can be seen from the analysis that the greatest difference between two CdZnTe detectors lies in the concentrations of deep hole traps especially T4, which is (9.41 ± 0.9) × 10^12^ cm^−3^ for the detectors with poor counting property and (8.97 ± 0.9) × 10^11^ cm^−3^ for the better detector. The difference is approximately one order of magnitude. For other defect levels, the difference between the two detectors is very small. It is seen that the main reason determining the performance of the detector is the trapping of holes, which forms an inverted built-in electric field and results in the distortion of the electric field within the detector. It is also shown from TSC test results that the detectors with poor counting properties have relatively high concentrations of deep hole traps.

### 2.2. Effect of Sub-Band-Gap Light Radiation on I–V Properties of CdZnTe Detectors

**Figure 2:**
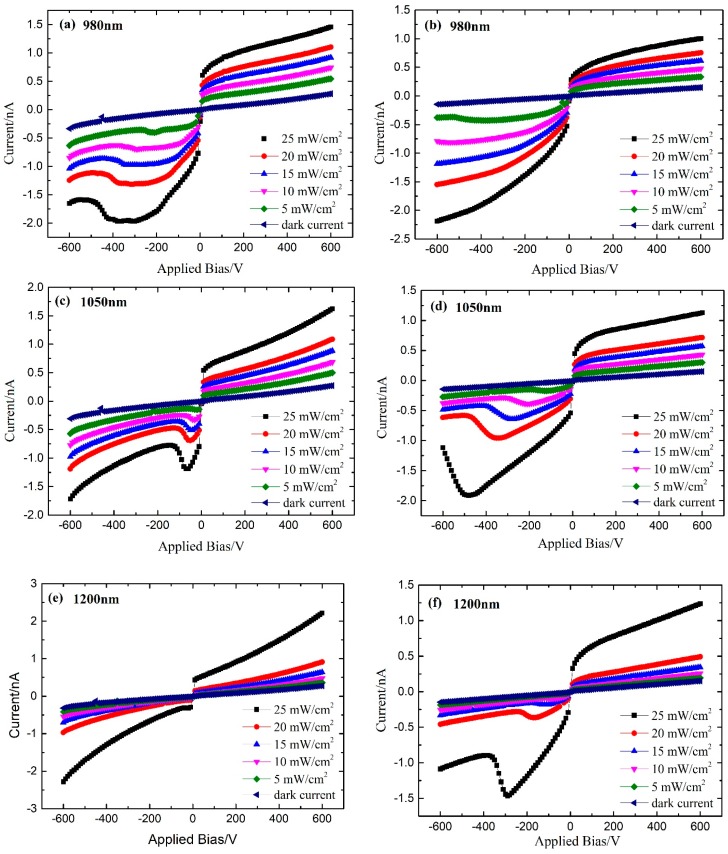

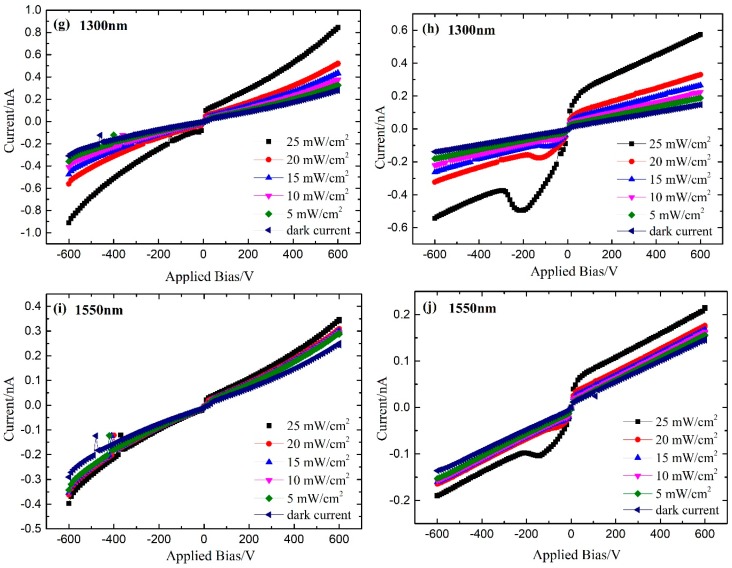
The current-voltage characteristics of CdZnTe detectors under radiation of the lights in the wavelength of 980 nm, 1050 nm, 1200 nm, 1300 nm, and 1550 nm without X-ray irradiation. (**a**,**c**,**e**,**g**,**i**) better counting performance detector; (**b**,**d**,**f**,**h**,**j**) poor counting performance detector.

The same CdZnTe detectors with different counting properties were selected to test the dark and light I–V curves under the radiation of different wavelengths and different intensity of sub-band-gap light of 980 nm, 1050 nm, 1200 nm, 1300 nm, and 1550 nm. The sub-band-gap light was irradiated from the side of cathode. The test temperature was 30 °C. The test results are shown in Figure 2, in which the dark I–V curves are measured without X-ray irradiation. It is seen that the photocurrents of CdZnTe detectors increase under the sub-band-gap light radiation of different wavelengths compared to the dark currents. For CdZnTe detectors with better counting properties, the operating bias and the amplitudes corresponding to the “inflection point” of the light I–V curve are much smaller than that with poor counting properties under the same wavelength and the same intensity. Considering the corresponding TSC test results, the deep hole trap concentrations in CdZnTe crystal with better counting properties are far less than that with poor counting properties. Therefore, the counting rates are higher under the same wavelength and the same intensity. Meanwhile, the probability of photo carrier trapping in the detector is small and charge accumulation is less likely to occur. The observed inflection point may be due to the trapping of free holes, which will increase the concentration of free electrons, thus leading to increase of photo-conductivity. Since the sub-band-gap light was irradiated from the side of cathode, where the stimulated charges are accumulated, the most severe variation of the current happened on the side of negative bias. 

For the same CdZnTe detector, the inflection point in I–V curve under the radiation of 1550 nm light corresponds to the lowest working bias. This indicates that CdZnTe crystal has very weak light absorption at this wavelength. With the decrease in the sub-band-gap light wavelength, the inflection point of the light I–V curve moves toward the higher working bias voltage, and the photocurrent value accordingly becomes larger. For the sub-band-gap light of the same wavelength, with the increase in light intensity, the photocurrent becomes larger, and the “knee” of the light I–V curve moves toward the high working bias direction. This implies that with the increase in the sub-band-gap light energy or the light intensity, the absorption of impurity defects in CdZnTe crystal increases, the photo carrier concentration increases, and the corresponding carrier capture probability increases.

### 2.3. X-ray Irradiation Tests of CZT Detectors with Poor Counting Properties

The purpose of introducing sub-band-gap light in CZT detectors was to improve the counting performance of the detectors with poor counting properties. Therefore, the detector with poor counting properties was further tested for the I–V curve with and without sub-band-gap light radiation under X-ray irradiation. We set the opt mechanical tube voltage to 80 kV, the tube current to 0.10 mA, the operating bias in the range from −600 to 600 V, and the control test system temperature to 30 °C. I–V curves of CdZnTe detectors irradiated under x-ray only and the one irradiated with different sub-band gap light wave length of 980 nm, 1050 nm, 1200 nm, 1300 nm, and 1550 nm lights were tested. 

**Figure 3:**
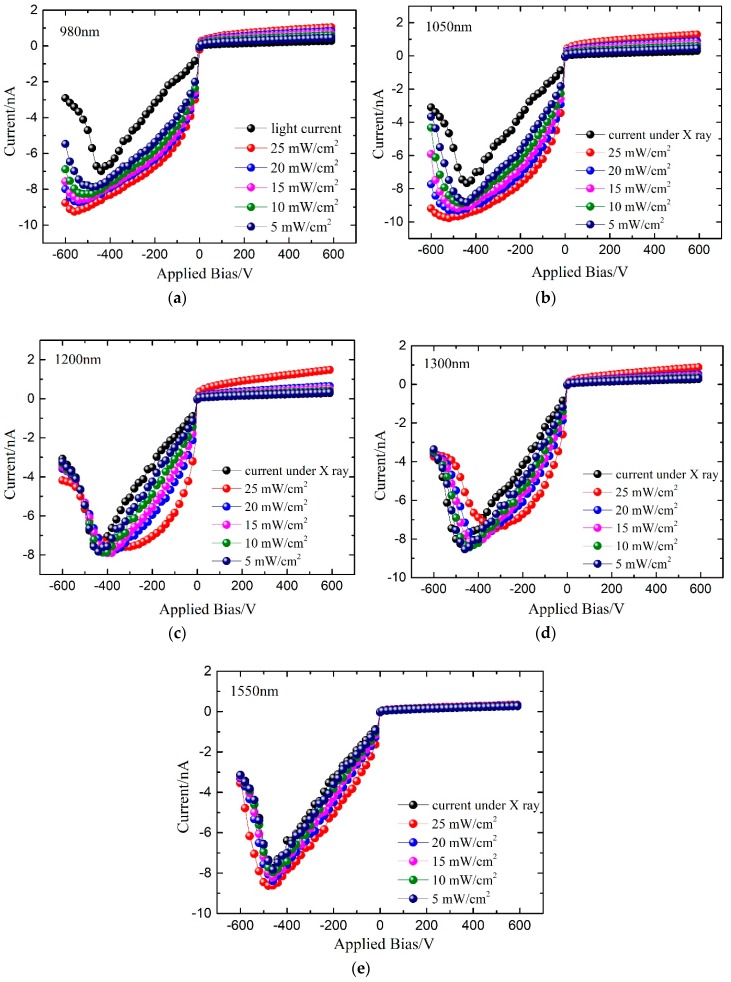
Photo I–V curves of CdZnTe detectors under the radiation of infrared light in the wavelengths of 980 nm (**a**), 1050 nm (**b**), 1200 nm (**c**), 1300 nm (**d**), and 1550 nm (**e**) with X-ray irradiation.

From the results shown in Figure 3, it can be seen that there are significant differences between I–V curves of the detector under sub-band-gap light of different wavelengths, and X-ray irradiation. For the same reason that the sub-band-gap light was irradiated on the side of cathode, the most significant change was observed for the negative bias side. When 1550 nm sub-band-gap light acts on a detector under X-ray irradiation, the light I–V curve hardly changes its position. Increasing the sub-band-gap light intensity produces no significant effect under the light of 1550 nm. 

For the case with the sub-band-gap light of 1200 nm and 1300 nm acting on a detector under X-ray irradiation in Figure 3, I–V curve moves in the direction of the low operating bias with respect to the position of the inflection point when no sub-band-gap light is applied. With the increase in the sub-band-gap light intensity, the position of the inflection point of the light I–V curve also moves toward the low working bias. But the sub-band-gap light of 1050 nm and 980 nm wavelengths produce the opposite effect. When they are applied to the detector under X-ray irradiation, the light I–V curve moves toward the high working bias voltage direction with respect to that without light. Increasing the light intensity will strengthen the position variation. The photocurrent value of the detector also increases significantly under 980 nm and 1050 nm sub-band-gap illumination. 

Because the inflection point in the light I–V curve marks the internal electric field distortion, the movement of the inflection point position of the light I–V curve represents the movement of the electric field distortion region. The shift of the inflection point toward a low operating bias voltage means that the electric field distortion will occur at a lower bias voltage, that is, the degree of electric field distortion under the originally operating bias weakens or even disappears. The sub-band-gap light of the wavelengths 1200 nm and 1300 nm decrease the electric field distortion in the detector. Stronger light intensity results in more obvious effects. The light at 980 nm and 1050 nm exacerbates the distortion of the internal electric field of the detector. The higher light intensity produces severe distortion of the internal electric field. However, the radiation with 1550 nm sub-band-gap light has almost no effect on the distortion of the internal electric field.

Considering the I–V results, the sub-band-gap light energy of 0.8005 eV (1550 nm) has no significant influence on the photoelectric performance of the detector. The sub-band-gap light of 0.9544 eV (1300 nm) and 1.034 eV (1200 nm) can effectively improve the electric field distortion because this sub-band-gap light can excite the vacant electrons trapped in the valence band and the trap holes. When the sub-band-gap light energy increases to 1.182 eV (1050 nm), the light radiation strengthens the electric field distortion in the detector, and increases the deterioration. This is because the sub-band-gap light of this energy can excite electrons in the defect level to enter the conduction band, i.e., increase the effective hole trap concentration, and increase the carrier concentration. The bulk holes accumulating near the cathode and the built-in electric field increase.

### 2.4. The Effects of Infrared Light Radiation on the X-ray Detection Performance of CZT Detectors

**Figure 4:**
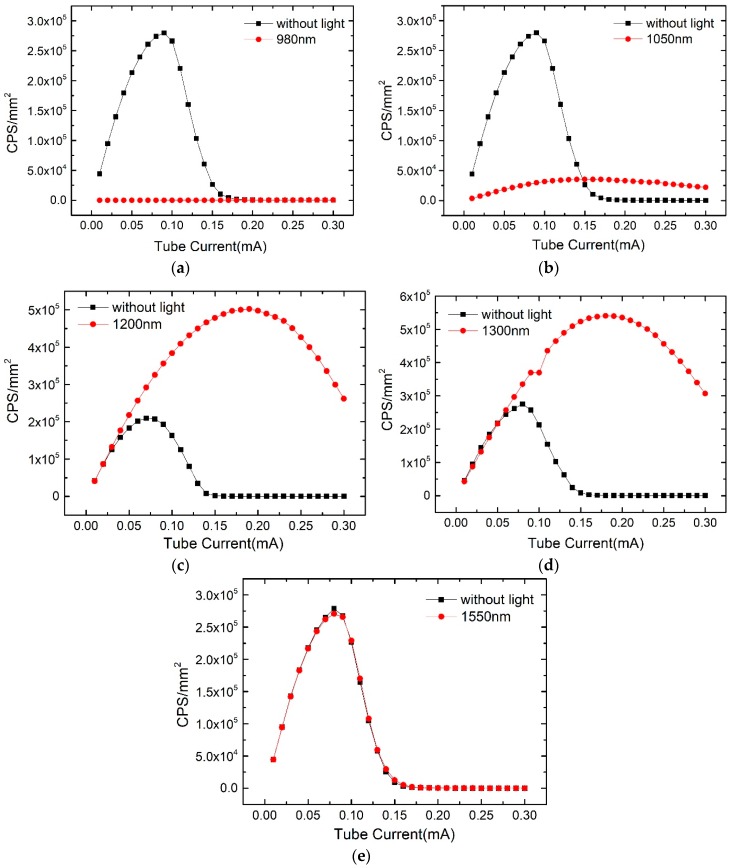
Variations in the count rate under light radiation of the wavelengths of 980 nm (**a**), 1050 nm (**b**), 1200 nm (**c**), 1300 nm (**d**) and 1550 nm (**e**).

The count rates of the detectors with poor counting properties were tested under different X-ray doses, and simultaneously radiated with the light of 980 nm, 1050 nm, 1200 nm, 1300 nm, and 1550 nm from an infrared LED lamp. The X-ray tube voltage was set to 80 kV, the tube current range was increased from 0.01 mA to 0.3 mA, and the bias voltage was kept at −500 V. The infrared lights irradiate from the side of CdZnTe detector, collimated with a copper shield. The test results given in Figure 4 show that the influence of the sub-band-gap on the counting rates of the detector is quite different for different wavelengths. The lights of 980 nm and 1050 nm degrades the counting rates of the CdZnTe detector, especially for the light of 980 nm, where the counting rate is almost reduced to 0, so the detector is unable to work normally. When using the light of 1050 nm, the count curve changed from the original highest count rate of 0.26 Mcps/mm^2^ to the lowest count rate of 0.04 Mcps/mm^2^. The light of 1200 nm and 1300 nm, in the middle of these 5 wavelengths, significantly improved the counting rates. At 1200 nm light, the maximum count rate of the detector was increased from the original 0.21 Mcps/mm^2^ to 0.5 Mcps/mm^2^, and the percentage increase is 138%. At 1300 nm light, the maximum count rate of the detector was increased from the original 0.25 Mcps/mm^2^ to 0.55 Mcps/mm^2^, and the percentage increase is 120%. For the light of 1550 nm, the count rate has nearly no change.

## 3. Conclusions

(1) The two CZT detectors with different counting rates selected for the studies were characterized with TSC. The major difference between the two detectors was their different concentrations of deep hole traps. The detector with higher counting rates had about one order higher deep hole trap concentration than that of the poor counting detector.

(2) Under different wavelengths of sub-band-gap light radiation, the current values of both the detectors increased and showed an inflection point in the I–V curve. For the same wavelength and same intensity of the sub-band-gap light, the counting rate is much higher and the working bias is considerably lower for the detector with lower deep hole trap concentration than that with higher deep hole trap concentration.

(3) The position of the inflection point was almost unchanged when the light wavelength was below 1550 nm. Sub-band-gap light radiation between 1200 nm and 1300 nm will make the inflection point move toward the low-bias direction, which is beneficial in weakening the electric field distortion. But 1050 nm and 980 nm sub-band-gap light radiation will make the inflection point move toward the high working bias voltage and distort the internal electric field more severely.

(4) The detector absorption for 1550 nm sub-band-gap light was very weak, therefore had no significant effect on X-ray detection performance. However, 1300 nm and 1200 nm sub-band-gap light will cause the recombination of valence band electrons with the holes trapped at the defect level, reduce the hole accumulation and weaken the built-in electric field. The light with the wavelength less than 1050 nm could excite electrons in the energy level of the defect into the conduction band, increase the effective hole trap concentration, and increase hole accumulation.

## Figures and Tables

**Table 1:** Summary of the main trap levels of two types of detectors.

	T1	T2	T3	T4	T5
Trap position (activation energy) (eV)	0.05 ± 0.01	0.11 ± 0.02	0.18 ± 0.03	0.43 ± 0.08	0.58 ± 0.1
Trap concentration of Figure 1a (cm^−3^)	(1.56 ± 0.2) × 10^13^	(1.02 ± 0.1) × 10^13^	(2.28 ± 0.2) × 10^12^	(8.97 ± 0.9) × 10^11^	(1.39 ± 0.1) × 10^12^
Trap concentration of Figure 1b (cm^−3^)	(2.88 ± 0.3) × 10^13^	(1.85 ± 0.2) × 10^13^	(3.09 ± 0.3) × 10^12^	(9.41 ± 0.9) × 10^12^	(2.96 ± 0.3) × 10^12^
